# Canonical WNT Signaling Inhibits Follicle Stimulating Hormone Mediated Steroidogenesis in Primary Cultures of Rat Granulosa Cells

**DOI:** 10.1371/journal.pone.0086432

**Published:** 2014-01-17

**Authors:** Andrea D. Stapp, Belinda I. Gómez, Craig A. Gifford, Dennis M. Hallford, Jennifer A. Hernandez Gifford

**Affiliations:** 1 Department of Animal Science, Oklahoma State University, Stillwater, Oklahoma, United States of America; 2 Department of Animal and Range Sciences, New Mexico State University, Las Cruces, New Mexico, United States of America; Nebraska Medical Center, United States of America

## Abstract

Beta-catenin (CTNNB1), a key component of wingless-type mouse mammary tumor virus integration site family (WNT) signaling, participates in follicle stimulated hormone-mediated regulation of estrogen (E_2_) production. The purpose of these studies was to determine if CTNNB1’s contribution to FSH-mediated steroidogenesis in primary rat granulosa cells was due in part to extracellular stimulation of the canonical WNT signaling pathway. To achieve this purpose, primary cultures of rat granulosa cells were exposed to vehicle or a canonical member of the WNT signaling pathway, WNT3A, before co-culture and in the presence or absence of FSH for 24 h. Activation of the canonical WNT signaling pathway was determined by dose-dependent induction of *Axin2* mRNA expression and stimulation of the CTNNB1/T cell factor promoter-reporter TOPflash. WNT pathway induction was demonstrated at doses of 50 and 500 ng/mL of WNT3A. Granulosa cells treated with WNT3A in combination with FSH had enhanced CTNNB1/T cell factor transcriptional activity above cells treated with WNT3A alone. Steroidogenic enzymes and ovarian differentiation factor mRNAs were quantified via quantitative PCR. Expression of steroidogenic enzyme mRNAs aromatase (*Cyp19a1*), P450 side chain cleavage (*Cyp11a1*), and steroidogenic acute regulatory protein (*Star*) were increased following FSH treatment. Co-incubation of WNT3A and FSH reduced the ability of FSH to stimulate steroidogenic enzymes and subsequent E_2_ and progesterone (P_4_) production. Concomitant activation of FSH and WNT pathways results in marked reduction of ovarian differentiation factors, LH receptor (*Lhcgr*) and inhibin-alpha (*Inha*). Therefore, WNT inhibits FSH target genes and steroid production associated with maturation and differentiation of the ovarian follicle.

## Introduction

The wingless-type mouse mammary tumor virus integration site (WNT) family of secreted glycoproteins participates in a variety of cellular processes including embryonic induction, axis specification, cell fate determination and differentiation [1], [2]. WNT ligands can activate three distinct intracellular signaling pathways which result in different biological activities. However, the most well studied WNT pathway is the canonical WNT signaling cascade which signals through the transcriptional co-factor, β-catenin (CTNNB1) to regulate target gene expression [3], [4]. Members of the canonical WNT signaling pathway are generally classified by their ability to transform mammary epithelial cell lines and include WNT-1,-2,-3A and -8 [5], [6]. Canonical WNTs are important in tissue homeostasis and recognized for their role in controlling cellular decisions to proliferate and differentiate [7]–[9]. However, mis-regulation of WNT/CTNNB1 signaling is linked to a range of pathologies including cancers of the breast, colon, and skin [2].

Activation of the canonical WNT signaling pathway requires a ternary complex composed of the WNT ligand bound to a seven transmembrane Frizzled (FZD) receptor and a low density lipoprotein receptor-related protein (LRP5/6) co-receptor. This interaction results in disruption of the multiprotein complex of adenomatous polyposis coli (APC), glycogen synthase kinase (GSK) 3-β and Axin responsible for phosphorylation and subsequent degradation of cytoplasmic CTNNB1. Stabilized cytoplasmic CTNNB1 accumulates and is translocated to the nucleus where it binds T-cell factor/lymphoid enhancer binding protein (TCF/LEF) to mediate transcriptional regulation by facilitating assembly of transcriptional co-activators such as CBP/p300 (cyclic AMP response element-binding protein) [10], legless (BCL9/LGS) and Pygopus [11]. Increased amounts of CTNNB1 restores transcription of TCF/LEF genes normally bound and repressed by complexes containing Groucho-related proteins [12]. Additionally, regulation of transcriptional activity by CTNNB1 occurs in part via functional interaction with steroidogenic factor-1 (NR5A1) [13]–[16].

The role of WNT signaling in the female gonad was first demonstrated in mice null for *Wnt4. Wnt4* deficient females exhibit partial sex reversal, with ovaries expressing genes associated with testis development and a paucity of oocytes at birth [17]. Subsequent work focused on the importance of WNT signaling molecules in the postnatal ovary. Multiple WNT and WNT family member transcripts exhibit stage specific expression within the adult ovary of rats, mice, and humans [18]–[21]. The *Wnt* family of genes has also shown to be hormonally regulated in adult ovaries. *Wnt4* expression is elevated in response to human chorionic gonadotropin and highly expressed in terminally differentiated luteal cells [18]. More recently, FSH has been shown to regulate *WNT2* mRNA expression in primary cultures of bovine granulosa cells [22]. The pattern of expression and the hormonal regulation of specific WNTs and FZDs detected in rodent ovaries indicate a role for WNT signaling in follicle maturation. Furthermore, CTNNB1 is required for maximal FSH and forskolin-stimulation of *Cyp19a1*, a regulation determined in a human granulosa cell line (KGN) to involve interaction with NR5A1 [13]. Subsequent studies using conditional deletion of CTNNB1 in primary mouse granulosa cell cultures demonstrated that a reduction in CTNNB1 compromised the ability of FSH to stimulate *Cyp19a1* and consequent estradiol production [23], further confirming *Cyp19a1* as a target of the CTNNB1 pathway in granulosa cells. While it has been reported that mice expressing constitutive activation of CTNNB1 in granulosa cells results in development of granulosa cell tumors [20], much remains unknown about the physiological significance of WNT/CTNNB1 in adult folliculogenesis.

The objective of this study was to investigate contribution of the canonical WNT signaling pathway in regulation of key ovarian steroidogenic enzymes and differentiation factors. Here, we report that co-incubation of canonical WNT3A with FSH results in an unexpected inhibition of steroidogenesis and genes known to be important for ovarian differentiation. We suggest canonical WNT signaling may be important to follicular maturation and potentially be identified as a new inhibitory pathway for follicle development through WNT negative feedback on TCF responsive genes.

## Materials and Methods

### Granulosa cell culture

All procedures involving animals were approved by the Oklahoma State University Institutional Animal Care and Use Committee (AG-10-3). Female Sprague-Dawley rats (17–21 days old) were purchased from Charles River Laboratories (Hollister, CA, USA) and housed within the Animal Resources Unit at Oklahoma State University with *ad libitium* access to feed and water. At 21–25 days, rats were injected subcutaneously for 3 consecutive days with 0.1 mL of 1.5 mg/mL 17 β-estradiol in propylene glycol [24]. Ovaries were harvested and trimmed to remove the bursa, fat, and oviducts, and incubated for 30 min at 37°C in 5% CO_2_ and 95% air, in 6 mM Ethylene glycol-bis(2-aminoethylether)-N,N,N’,N’-tetraacetic acid in Dulbecco's Modified Eagle Medium/Ham's F-12 (Invitrogen, Grand Island, NY, USA) supplemented with 1% (v/v) 100 IU/mL penicillin/100 µg/mL streptomycin (DMEM/F12/PS) medium. Ovaries were then incubated for 30 min in 0.5 M sucrose in DMEM/F12/PS. Granulosa cells were mechanically isolated from ovaries by penetration of follicles with a 30-gauge needle. Cell number and viability were determined via hemocytometer using trypan blue exclusion. Granulosa cells were plated (1.4 –1.8×10^6^ per 60-mm tissue culture dish) in DMEM/F12/PS medium supplemented with 10% (v/v) fetal bovine serum (Invitrogen) and allowed to attach for 24 h at 37°C in 5% CO_2_, 95% air before treatment. For WNT3A dose response experiments, medium and unattached cells were aspirated and granulosa cells were exposed to 1, 5, 50, or 500 ng/mL recombinant mouse WNT3A (R&D Systems, Minneapolis, MN, USA; WNT3A) or phosphate buffered saline (PBS) + 0.1% bovine serum albumin (BSA; Fisher Scientific, Fair Lawn, NJ, USA) in serum-free DMEM/F12/PS medium for 24 h. Doses of WNT3A were chosen based on manufacturer’s product sheet information indicating an ED_50_ of ≤ 51ng/mL in HEK293T human embryonic kidney cells, and preliminary studies in our lab demonstrating 50 ng/mL as the lowest dose capable of inducing WNT signaling in primary rat GC. Immediately following WNT3A treatment, GC were treated with 100 ng/mL purified human FSH (S1AFP-B-3; National Hormone and Peptide Program, National Institute of Diabetes and Digestive and Kidney Diseases, National Institutes of Health, Bethesda, MD, USA) or PBS prepared in serum-free medium supplemented with 10^−7^ M testosterone propionate. FSH or PBS treatments were added directly to cell media and cells were incubated for 24 h. A subsequent time course experiment was performed in which cells were co-treated with FSH for 24 h and 50 ng/mL of WNT3A for a total of 24, 30, 36 or 48 h. Complete medium was removed from all plates and replaced with incomplete media containing the vehicle control or 50 ng/mL of WNT3A at designated time points prior to FSH treatment. Follicle stimulating hormone treatment was performed as previously described. Treatments for all experiments were terminated 24 h following FSH treatment by removing medium and rinsing cells once with ice cold PBS. Cells were scraped into 1 mL TRIzol (Invitrogen) reagent and stored at –80°C until isolation of RNA and protein.

### RNA extraction and reverse transcription PCR

RNA was isolated from cultured granulosa cells using TRIzol reagent according to the manufacturer’s protocol and stored at –80°C. Integrity of RNA was assessed by visualization of 18S and 28S ribosomal RNA resolved by agarose gel electrophoresis. RNA purity and quantity was determined using a NanoDrop ND-1000 Spectrophotometer (Thermo Fisher Scientific, Wilmington, DE, USA). Purity was determined by 260/280 nm absorbance ratios, absorbance ratios above 1.8 were considered acceptable. Total RNA (1 µg) was treated with 1 µL DNase I (Invitrogen) to remove genomic DNA contamination following manufacturer’s instructions. First-strand cDNA was synthesized from total RNA using oligo(dT) primers and 1 µL Superscript II Reverse Transcriptase (Invitrogen). Samples were stored at –20°C until analysis. All gene specific primers were designed using Primer3 [25] and synthesized by Integrated DNA Technologies (Coralville, IA, USA). Forward and reverse primer sequences are listed in Table 1. Primers were optimized for appropriate primer concentration using a concentration gradient (150, 200, 250, 300, and 350 nM) and validated using a 7-log dilution curve as previously reported [26].

**Table 1:** Primer sequences used in real-time PCR.

		Sequences of primers (5′- 3′)	
Gene	Accession no.	Forward	Reverse
1 *Actb*	NM_031144	TATGGAATCCTGTGGCATCC	CTTCTGCATCCTGTCAGCAA
2 *Axin2*	NM_024355	CTGGCTATGTCTTTGCACCA	AGGAGGGATTCCATCTACGC
3 *Cyp11a1*	NM_017286	CTATGCCATGGGTCGAGAAT	CAGCACGTTGATGAGGAAGA
4 *Cyp19a1*	NM_017085	TAACAACAACCCGAGCCTGT	GTGTCTCATGAGGGTCACCA
5 *Gapdh*	NM_017008	ATGACTCTACCCACGGCAAG	TACTCAGCACCAGCATCACC
6 *Inha*	NM_012590	CTTATGTATTCCGGCCATCC	AGAGCTATTGGAGGCTGCTG
7 *Lhcgr*	NM_012978	ATGCCATCCCAATTATGCTC	AGGCAGATGCTGACCTTCAT
8 *Mrpl19*	NM_001029898	GTGCCTGTGAACAAGCTGAA	CATGGCTTGCTCCACTTCTG
9 *Ppia*	NM_017101	AGCATACAGGTCCTGGCATC	CCATCCAGCCACTCAGTCTT
10 *Star*	NM_031558	CACAGTCATCACCCATGAGC	AGGTGGAACCTCTACGCTTG

^1^ *Actb*  =  actin-beta.

2 *Axin2*  =  axin inhibition protein 2.

3 *Cyp11a1*  =  P450 side chain cleavage.

4 *Cyp19a1*  =  aromatase.

5 *Gapdh*  =  glyceraldehyde 3-phosphate dehydrogenase.

6 *Inha*  =  inhibin-alpha.

7 *Lhcgr*  =  luteinizing hormone chorionic gonadotropin receptor.

8 *Mrpl19*  =  mitochondrial ribosomal protein L19.

9 *Ppia*  =  peptidylprolyl isomerase A.

10 *Star*  =  steroidogenic acute regulatory protein.

### Quantitative real-time PCR

A working solution of cDNA was prepared by diluting samples 1∶10 with DEPC-treated water. Five microliters of cDNA working solution was added to a 25 µL master mix containing 13 µL SYBR green and fluorescein mix (Bioline, Taunton, MA, USA), and 0.5 – 0.875 µL of each forward primer (10 µM) and reverse primer (10 µM). Quantitative real-time PCR analysis was carried out using a Bio-Rad MyiQ single color real-time PCR detection system and MyiQ software (Bio-Rad Laboratories, Hercules, CA, USA). Standard thermocycler conditions were as follows: 95°C for 10 min, followed by 40 cycles of 95°C for 15 sec, 60°C for 30 sec, and 72°C for 30 sec. Relative fold change in target mRNAs was quantified using the ΔΔCq method where ΔΔCq was determined by subtracting the average control ΔCq from the ΔCq of the sample [27]. All reverse-transcribed cDNA samples were assayed in duplicate for each gene, and melt curve analyses were performed to ensure specificity of amplification. Melt curve analysis was carried out for 81 cycles with 0.5°C temperature increase from 55°C to 95°C.

To determine the appropriate reference gene to normalize cDNA variability between samples, a panel of four reference genes were analyzed including, glyceraldehyde-3-phosphate dehydrogenase (*Gapdh*), β-actin (*Actb*), peptidylprolyl isomerase A (*Ppia*), and mitochondrial ribosomal protein L19 (*Mrpl19*). The raw Cq values were obtained for each gene in all samples and analyzed using GeNorm (Biogazell qbasePLUS2, Zwijnaarde, Belgium) to determine the most stable normalization factor. The most stable housekeeping gene for target gene normalization was determined to be *Mrpl19* and was used as the reference gene for the experiments [28].

### Transient transfections and luciferase assay

Primary rat granulosa cells (1.5×10^6^/well in 24-well plate) and KGN (1.69×10^6^/well in 24-well plate) were plated in complete medium to achieve 60% confluency prior to transfection. Each treatment was performed in duplicate in three separate experiments. Transient transfections were performed using Lipofectamine LTX and Plus Reagent (Invitrogen, Carlsbad, CA) following manufacturer’s specifications. Briefly, cells were transfected with 200 ng/well of TOPflash TCF Reporter Plasmid (Upstate Cell Signaling, Lake Placid, NY). All groups were cotransfected with Renilla luciferase reporter vector to normalize for differences in transfection efficiency. Cells were exposed to lipofectamine mixture for ∼6 h in serum free conditions, after which complete medium was added and the cells were allowed to recover for 18 h. Cells were co-treated with WNT3A and FSH as previously described. Twenty-four hours after treatment cells were harvested using 1x Passive Lysis Buffer (Promega, Madison, WI). Luciferase assays were performed using the Dual-Luciferase Reporter Assay System (Promega) and a Modulus Luminometer (Turner Biosystems; Sunnyvale, CA).

### Western blotting

Protein was isolated from the primary granulosa cells collected in Mammalian Protein Extraction Reagent (Thermo Scientific, Rockford, IL, USA), according to the manufacturer’s protocol. Protein concentrations were estimated using a BCA Protein Assay Kit (Thermo Scientific). Total protein lysates were separated by 10% SDS-PAGE Tris-HCl gels and resolved proteins were transferred to polyvinylidene fluoride (PVDF) membranes (Bio-Rad Laboratories) at 4°C. The PVDF membranes were blocked at room temperature for 1 h in Tris buffered saline containing 5% nonfat dry milk (Nestle Carnation, Solon, OH, USA) and 0.1% Tween-20 (Sigma-Aldrich, St. Louis, MO, USA). Western blot analysis for CTNNB1 was performed using anti-CTNNB1 (BD Transduction Laboratories, San Diego, CA, USA) at a concentration of 1∶10,000. Following incubation with primary antibody, the membrane was incubated with horseradish peroxidase-conjugated secondary antibody (1∶10,000; Thermo Scientific). Antigen-antibody complexes were detected by chemiluminescence with Immobilon detection reagent (Millipore, Billerica, MA, USA). Protein loading was assessed by reprobing membranes for β-actin (Cell Signaling Technology, Danvers, MA, USA) at a final concentration of 1∶1,000, followed by incubation with HRP-conjugated secondary antibody at a concentration of 1∶3,000. Quantification was carried out using the AlphaEaseFC image acquisition system (Alpha Innotech, Santa Clara, CA, USA).

### Radioimmunoassay

Granulosa cell culture media was analyzed for E_2_ and P_4_ by solid-phase radioimmunoassay using components of Siemens Medical Diagnostics Corp (Los Angeles, CA, USA) commercial kits as previously described [22]. The E_2_ concentration in samples of cell culture media was determined in 200 µL of media and the specific binding was 66%. Detection limit (95% of maximum binding) of the assay was 2 pg/mL. Intra-assay CV for E_2_ was 6.2% for cell culture media.

The P_4_ concentration in samples of granulosa cell media was assayed at 100 µL. The specific binding was 56%. Detection limit (95% of maximum binding) of the assay was 10 pg/mL. Intra-assay CV for P_4_ was 4.9% for cell culture media.

### Statistical analysis

Experiments were analyzed for a completely randomized design in which four treatments were included; control, FSH, WNT3A, and WNT3A plus FSH. Five independent replicates for each treatment were used to analyze relative changes in gene expression for *Cyp19a1*, *Axin2*, *Cyp11a1*, *Inha*, *Lhcgr*, *Star*, *Mrpl19*, and three independent replicates for TOPflash promoter-reporter assay and medium hormonal concentration of P_4_ and E_2_. Data were analyzed using general linearized mixed model with fixed effects of WNT and FSH. Means were compared using LSD comparisons and separated using linear and quadratic contrasts. Differences in relative protein abundance of CTNNB1 in three replicate experiments were analyzed using the GLM procedures of SAS. All tests of significance were performed at the 0.05 level of significance. All data analysis was computed using SAS/STAT software, SAS® version 9.3 (SAS Institute, Cary, NC, USA).

## Results

### The frizzled receptor agonist WNT3A, induces the canonical WNT signaling pathway in granulosa cells

**Figure 1:**
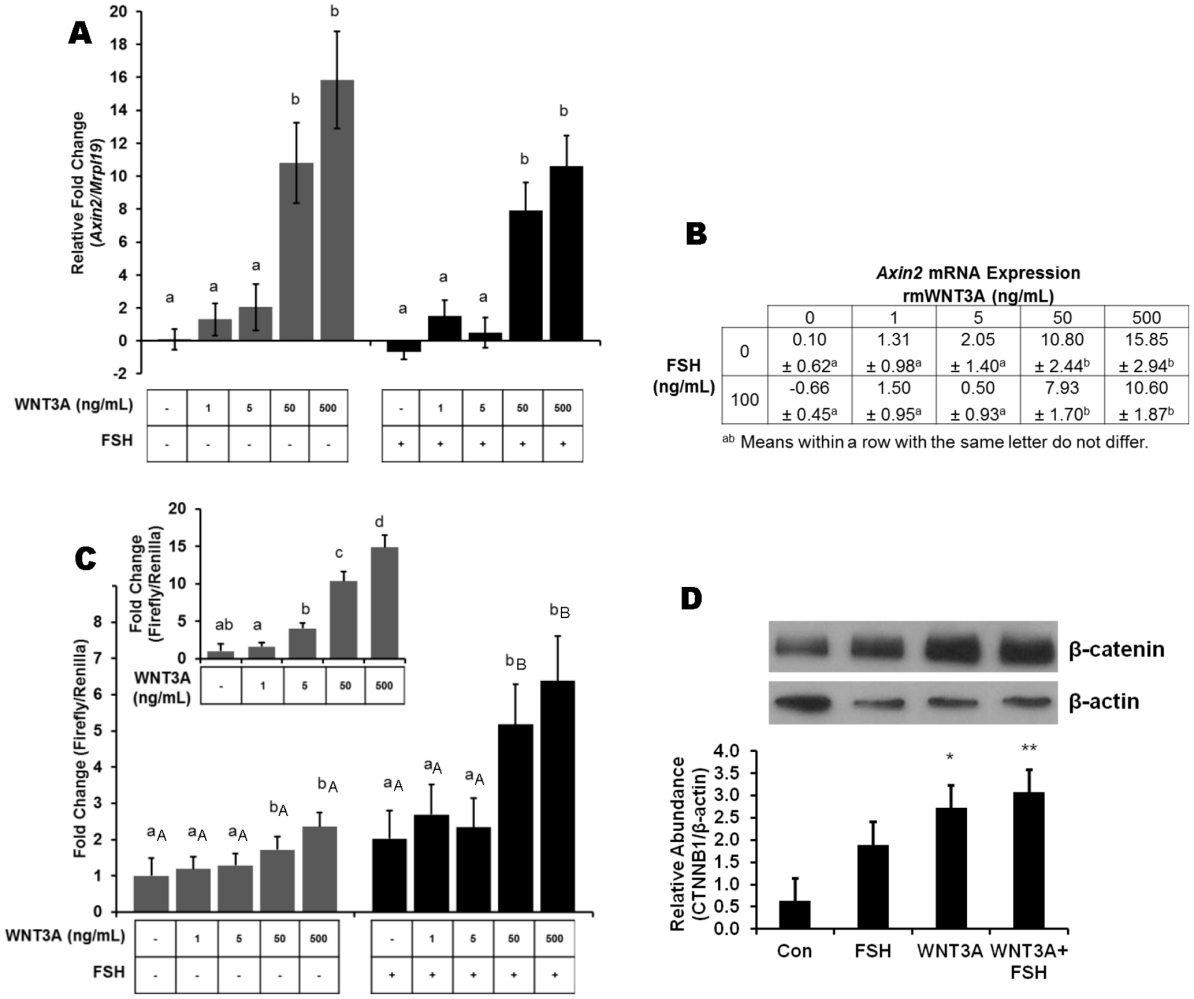
Induction of the WNT signaling pathway. Specific induction of WNT signaling is demonstrated by regulation of the WNT target gene, *Axin2,* transcriptional regulation of CTNNB1/TCF luciferase reporter and the transcriptional co-factor CTNNB1. Primary rat granulosa cells were treated for 24 h with vehicle or increasing doses of recombinant WNT3A (1, 5, 50, or 500 ng/mL) in the presence or absence of FSH (100 ng/mL). (A) Graphical representation of *Axin2* mRNA expression and (B) tabular representation of *Axin 2* mRNA expression data analyzed by real-time PCR (n  =  5). (C) CTNNB1/TCF-dependent (TOPflash) transcriptional activity in primary GC (n  =  3) and a GC tumor cell line (inset, n  = 3). Statistical significance is presented as the mean ± standard error of the mean with significance set at (*P*<0.05). Results of WNT treatment are compared within experimental groups incubated without (grey bars) and with (black bars) FSH treatment. Means with the same letter do not differ significantly. (D) Representative Western blot and quantitative analysis (n  =  3) of CTNNB1 protein demonstrates an increase in CTNNB1 abundance after treatment with WNT3A alone (*P*<0.05) and co-treatment of WNT3A + FSH (*P*<0.01) compared with controls.

WNT3A is a canonical WNT expressed in postnatal ovaries of mice [29], and the presence of WNT3A in bovine granulosa cells suggests a role in ovarian function [30], [31]. Activation of the WNT signaling pathway by WNT3A treatment was evaluated by quantification of *Axin2* mRNA, a direct target of WNT signaling which is initially induced upon WNT stimulation and subsequently acts as a negative feedback mechanism to restrict the duration of signal [32]–[34]. Low concentrations of WNT3A (1 and 5 ng/mL) were unable to stimulate the WNT signaling pathway; however, WNT3A at 50 and 500 ng/mL increased endogenous *Axin2* mRNA 10 and 15-fold greater, respectively, than control or FSH treatment groups (Fig. 1A-B; *P*<0.01). To assess whether WNT3A regulates CTNNB1/TCF-mediated transcription, TOPflash luciferase reporter assays were conducted in a granulosa tumor cell line (KGN) and in primary rat granulosa cells. Primary rat granulosa cells treated with 50 and 500 ng/mL of WNT3A responded with approximately 2-fold increase in TOPflash activity. A similar pattern of promoter/reporter activity was demonstrated in KGN cells, albeit to greater levels of activation (Fig. 1C, inset). FSH potentiated WNT3A induction of TOPflash in cells stimulated with 50 or 500 ng/mL of WNT3A compared to control or FSH treatment groups (Fig. 1C; *P*<0.05). Consistent with these results, Western blot analysis showed that CTNNB1 protein accumulation in granulosa cells was increased following stimulation with WNT3A (*P*<0.05) and co-treatment of WNT3A plus FSH (*P*<0.01) compared with controls (Fig. 1D). These data indicate functional activation of the canonical WNT signaling pathway by WNT3A at concentrations between 50 and 500 ng/mL.

### Stimulation of the WNT signaling pathway alters FSH-mediated gene expression in primary rat granulosa cells

A preliminary study was conducted to determine if duration of WNT3A treatment differentially affected gene transcription or steroid production. Granulosa cells were incubated with WNT3A for 24, 36 or 48 h alone or in combination with a 24 h FSH treatment. Time point selection was determined based on previous studies which commonly evaluate WNT signaling 24 to 48 h post WNT stimulation [35]–[37], and because maximal stimulation of FSH regulated genes is demonstrated at 24 h. Two highly repeatable experiments demonstrated similar fold-induction of *Axin2* mRNA expression among the different time points (data not shown). Additionally, mRNA expression of steroidogenic enzymes and steroid production showed a regulation that was dependent on treatment but independent of treatment timeline (data not shown). Therefore, for subsequent experiments cells were treated with WNT3A and FSH simultaneously and allowed to incubate for 24 h prior to analysis. Consistent with the detailed experiments below, stimulation of both WNT and FSH signaling pathways markedly reduced the ability of FSH to stimulate expression of ovarian derived steroidogenic enzymes and resultant steroidogenesis.

**Figure 2:**
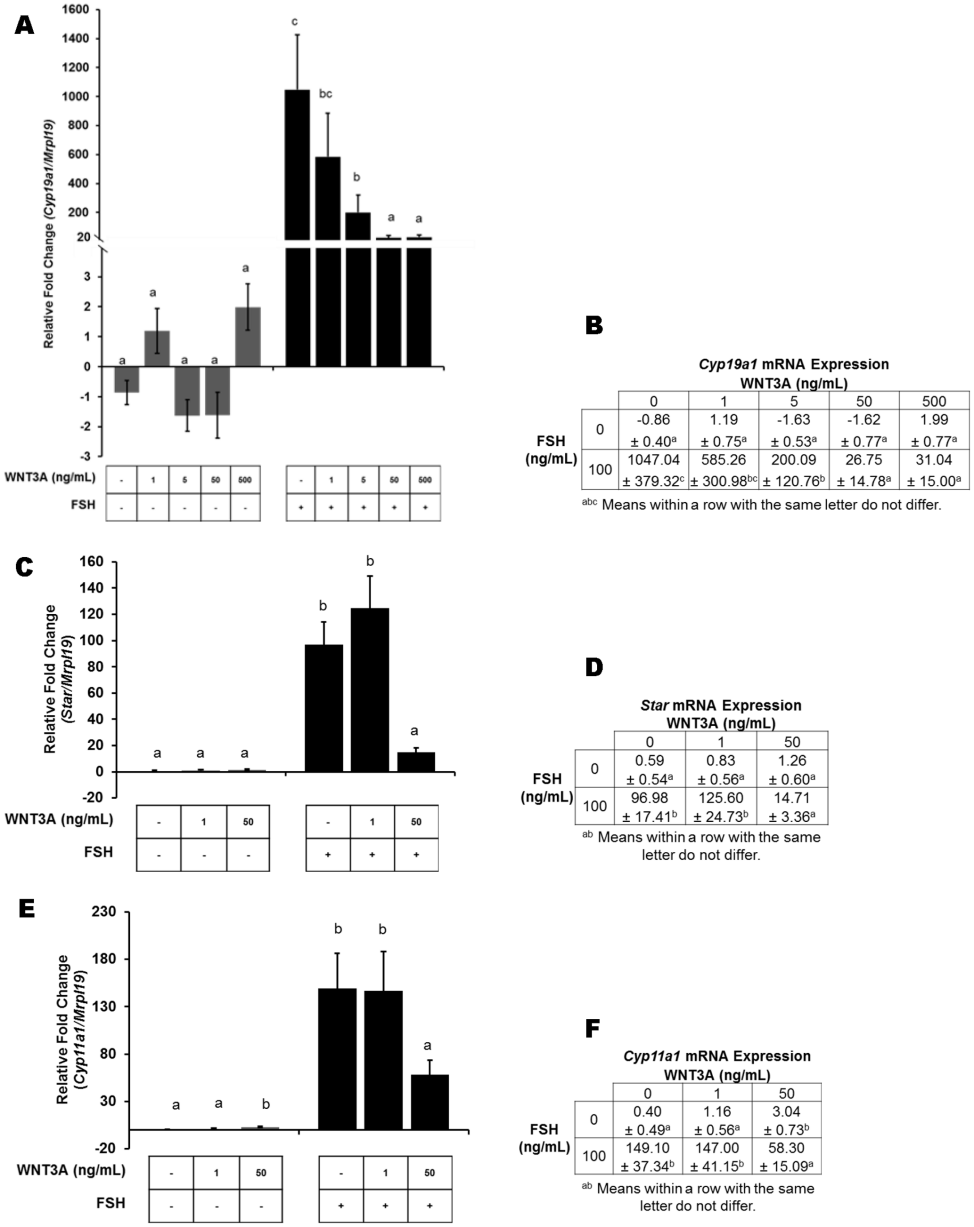
WNT signaling inhibits expression of FSH target genes. FSH induced expression of key steroidogenic enzymes is inhibited by stimulation of canonical WNT signaling. Quantitative PCR analysis of key steroidogenic enzyme mRNAs in rat granulosa cells treated as indicated in Figure 1. Graphical fold change (A) and (B) tabular representation of *Cyp19a1* mRNA expression demonstrate WNT3A at 50 and 500 ng/mL mutes FSH mediated *Cyp19a1* expression. (C-D) *Star*, and (E-F) *Cyp11a1* mRNA expression in cultured rat granulosa cells was evaluated at 1 and 50 ng/mL recombinant WNT3A followed in the presence or absence FSH. Similar to expression of *Cyp19a1* mRNA, FSH-induced expression of *Star*, and *Cyp11a1* above vehicle treated controls and all WNT3A treated cells. Co-stimulation with both FSH plus 50 ng/mL WNT3A reduced FSH mediated gene transcription. Gene expression is presented as the mean ± standard error of the mean (n  =  5) with significance set at *P*<0.05. Results of WNT treatment are compared within experimental groups incubated without (grey bars) and with (black bars) FSH treatment. Means with the same letter do not differ significantly.

To determine whether WNT signaling contributes to stimulation of FSH target genes, mRNA expression for key steroidogenic enzymes was evaluated. Preantral granulosa cells treated with FSH demonstrated an upregulation (*P*<0.01) of *Cyp19a1* mRNA compared to vehicle treated controls or cells cultured with increasing doses of WNT3A, indicating specific induction of FSH signaling (Fig. 2A). In contrast, a WNT and FSH interaction was detected and WNT effects were only revealed when FSH was present. A decreasing quadratic trend was evident in cells co-stimulated with 5, 50, or 500 ng/mL WNT3A and FSH (*P*<0.05) resulting in inhibition of FSH’s ability to induce *Cyp19a1* mRNA. Since data in Figure 1 indicates that 1 ng/mL of WNT3A was not sufficient to activate WNT signaling and 50 ng/mL was the lowest sufficient dose for extracellular activation of the canonical WNT signaling pathway, analysis of key steroidogenic enzymes was evaluated at both doses. Consistent with the results for *Cyp19a1*, upregulation of mRNAs encoding steroidogenic acute regulatory protein (*Star*) and cytochrome P450 side-chain-cleavage (*Cyp11a1*) were restricted to granulosa cells treated with FSH (Fig. 2C and 2E). The addition of WNT3A at 50 ng/mL, but not at 1 ng/mL, consistently compromised the ability of FSH to induce *Star* (Fig. 2C-D; *P*<0.01) and *Cyp11a1* (Fig. 2E-F; *P*<0.05) mRNA expression.

### Exogenous WNT3A inhibits FSH mediated granulosa cell steroidogenesis

**Figure 3:**
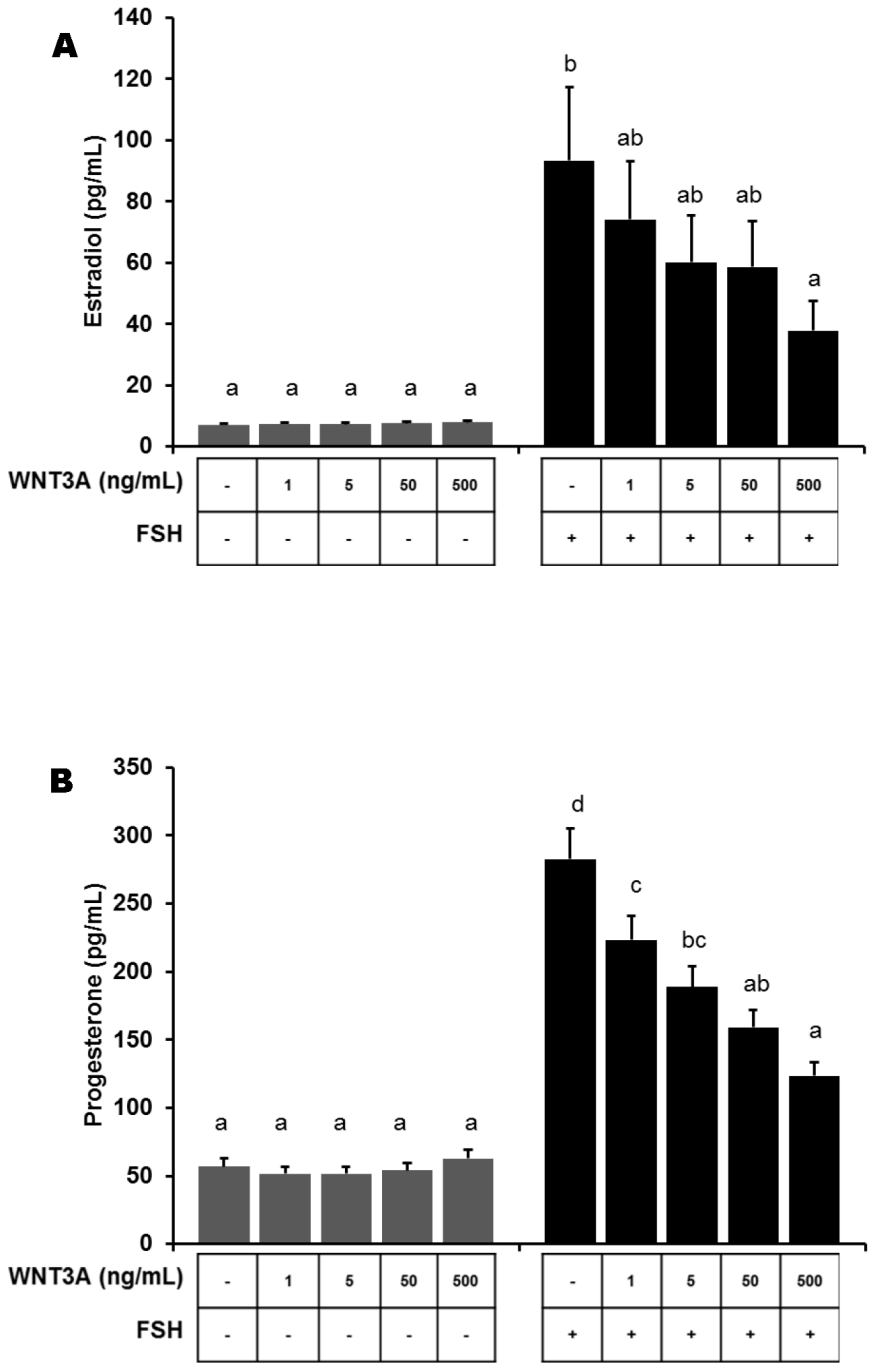
FSH stimulation of P_4_ and E_2_ is reduced with co-incubation of WNT3A and FSH. Primary rat granulosa cells were treated as described in Fig. 1. Estradiol and progesterone concentrations (n  =  3) in cell culture medium were analyzed by RIA. (A) FSH treatment increased (*P*<0.01) media E_2_ concentrations compared to controls. FSH stimulation of E_2_ was markedly decreased (*P*  =  0.05) in cells exposed to both FSH plus 500 ng/mL WNT3A. (B) Media P_4_ concentrations increased (*P*<0.01) in FSH-treated granulosa cells compared with control; the stimulatory effect of FSH on P_4_ synthesis was reduced (*P*<0.05) with increasing concentrations of WNT3A. Statistical significance is presented as the mean ± standard error of the mean with significance set at *P*<0.05. Results of WNT treatment are compared within experimental groups incubated without (grey bars) and with (black bars) FSH treatment. Means with the same letter do not differ significantly.

To determine whether WNT3A inhibition of FSH-regulated steroidogenic enzyme mRNAs also resulted in modulation of granulosa cell steroid production, media concentrations of estradiol (E_2_) and progesterone (P_4_) were examined. Following FSH stimulation, media concentrations of E_2_ were increased (*P*<0.01) compared to vehicle treated controls and cells treated with WNT3A. However, the stimulatory effect of FSH on E_2_ production was reduced (linear effect; *P*<0.05) in granulosa cells co-incubated with increasing doses (1, 5, 50 or 500 ng/mL) of WNT3A and 100 ng/mL of FSH (Fig. 3A). Similarly, media P_4_ concentrations increased 5.4-fold (*P*<0.01) in FSH-treated granulosa cells compared with control and WNT3A treatments (Fig. 3B). As was demonstrated for E_2_ production, the stimulatory effect of FSH on P_4_ synthesis was reduced (linear effect; *P*<0.01) by the presence of WNT3A.

### Granulosa cell differentiation factor transcripts show distinct regulation

**Figure 4:**
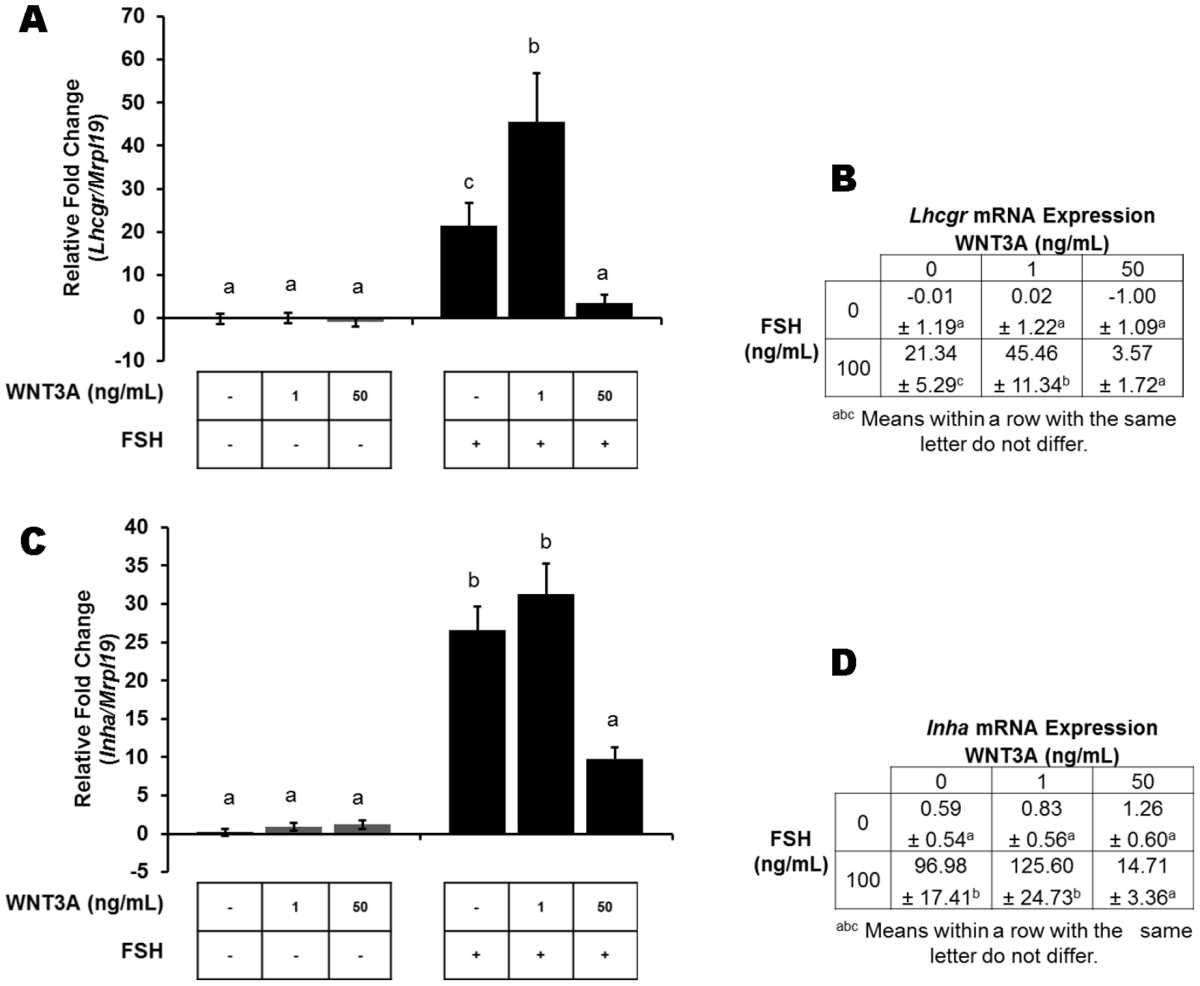
Canonical WNT signaling inhibits FSH-induced expression of ovarian differentiation factors. Fold change in *Lhcgr* and *Inha* mRNA was analyzed by real-time PCR. (A -B) WNT3A abrogated the response of *Lhcgr* and (C-D) *Inha* mRNA expression to FSH stimulation. Expression is presented as the mean ± standard error of the mean (n  =  5) with significance set at *P*<0.05. Results of WNT treatment are compared within experimental groups incubated without (grey bars) and with (black bars) FSH treatment. Means with the same letter do not differ significantly.

To further elucidate the participation of canonical WNT signaling on follicular maturation, gene expression for ovarian differentiation factors were quantified. Basal expression of LH receptor (*Lhcgr*), and inhibin alpha (*Inha*) mRNA was detected but not different in non-stimulated granulosa cells and cells treated with 1 or 50 ng/mL WNT3A. *Lhcgr* and *Inha* mRNA levels responded robustly to FSH treatment when compared to their respective vehicle-treated or 1 ng/mL WNT3A control (*P*<0.01; Fig. 4). However, the gonadotropin induction of *Lhcgr* and *Inha* expression with FSH alone was abrogated with co-stimulation of the WNT (50 ng/mL, WNT3A) and FSH extracellular pathways (Fig. 4A and 4C; *P*<0.01), a response similar to the steroidogenic enzymes.

## Discussion

The ability of FSH to facilitate maturation of ovarian follicles and synthesis of follicular E_2_ relies on the input from a variety of signaling molecules [38], [39]. Secreted WNT glycoproteins have been identified as regulators of ovarian cell function and follicular organization. The *Wnt* family of genes are hormonally regulated in rodent and bovine ovaries. In rodent ovaries, *Wnt4* expression is elevated in response to human chorionic gonadotropin and highly expressed in terminally differentiated luteal cells [40]. Likewise, we reported that bovine granulosa cells demonstrate an upregulation of *Wnt2* mRNA expression following FSH stimulation [22]. The canonical WNT pathway relies on activation of the downstream effector, CTNNB1 to transduce a signal. A requirement for CTNNB1 in FSH regulation of key steroidogenic enzymes has been identified in primary culture of rat granulosa cells [13], and reduction of CTNNB1 resulted in a compromised ability of FSH to stimulate *Cyp19a1* mRNA and subsequent E_2_ production [23]. Additionally, granulosa cells of large bovine antral follicles producing high amounts of E_2_ demonstrated an increase in CTNNB1 accumulation compared to low E_2_ producing follicles [22]. Collectively, these data suggest FSH and WNT signaling pathways may work together to impact steroid production in the postnatal ovary. Surprisingly, our data provide novel evidence indicating activation of canonical WNT signaling inhibits expression of FSH target genes associated with regulation of follicle maturation and steroid hormone production. Specifically, exogenous stimulation of primary granulosa cells with recombinant WNT3A effectively mutes the ability of FSH to regulate transcription of the steroidogenic enzymes *Cyp19a1, Star* and *Cyp11a1* and decreases production of both E_2_ and P_4_. WNT3A is a member of the canonical WNT family of secreted molecules and is recognized for its’ ability to stabilize CTNNB1 protein which accumulates and enters the nucleus to activate transcription of TCF/LEF target genes. WNT3A is expressed in postnatal ovaries of mice [29] and cattle [30] and therefore may be involved in regulating ovarian gene expression. An interaction between WNT signaling and G-protein coupled gonadotropin receptors is evident and appears to be dependent on stage-specific development of the ovarian follicle. The negative regulation of WNT signaling on gonadotropin stimulation of steroidogenic enzymes and ovarian differentiating factors is consistent with previous studies in which granulosa cells from mice expressing dominant stable CTNNB1 had reduced expression of *Lhcgr*, *Star*, and *Cyp11a1* following forskolin-induced cAMP activation and PMA-activated PKC signaling [22]. The suggestion by Fan et al. [41] that overactivation of CTNNB1 is responsible for the negative effects on LH-induced events is probable given our findings that the greatest observed increase in transcriptional activity of the CTNNB1/TCF responsive promoter (TOPflash) occurred in cells stimulated by WNT and FSH at doses capable of muting steroid synthesis. Our data show that WNT3A and FSH act synergistically to activate TOPflash, whereas WNT3A blunts the FSH response on other gene targets. These data highlight the importance of promoter context as TOPflash is an artificial minimal promoter composed of several TCF response elements. It is not likely that a minimal promoter will mimic native promoters that are composed of many different response elements. TOPflash is simply a positive control demonstrating WNT3A activity, especially when added with FSH.

Whereas previous studies indicate overexpressing CTNNB1 contributes to FSH induction of *Cyp19a1* expression in primary granulosa cell cultures of rodents [13], [42] and bovine [22], our data demonstrate WNT3A stimulation of CTNNB1 results in the downregulation of FSH target gene expression. We suggest a model in which FSH may be regulating expression of a canonical WNT that then establishes a negative feedback loop. This is supported by recent data in primary cultures of bovine granulosa cells demonstrating that FSH regulated expression of canonical *WNT2* [22]. Similar to the bovine, microarray analysis of rat granulosa cells treated with FSH noted induction of several WNT ligands [43] any of which might be involved in the negative feedback regulation. For our experiments, we utilized WNT3A as a surrogate for canonical WNT signaling since to our knowledge a biologically active WNT2 (a WNT expressed in postnatal granulosa cell and known to regulate CTNNB1/TCF signaling [44]) is not commercially available. Our co-treatment paradigm allowed for detection of the negative feedback mechanism. Induction of *Axin2* mRNA suggests that WNT3A induces an inhibitor that acts in a context-specific manner on promoters that respond to FSH. Therefore, even though WNT3A can activate CTNNB1, this positive effect must be overridden by another mechanism. Law et al. [45] shows that FSH can stimulate the phosphorylation of CTNNB1 via activation of PKA and that TCF mediates FSH-responsiveness of *Lhcgr.* We propose that FSH regulates expression of WNT(s) which sets up a negative feedback loop to control TCF responsive genes. This mechanism would ensure that CTNNB1 remains controlled so that TCF responsive genes are not overexpressed. Consistent with this notion is the fact that TCF family members contribute to expression of numerous FSH target genes in granulosa cells including *Cyp19a1*, *Inha*, *Foxo1, Lhcgr* and others [45].

Numerous alternative scenarios for regulation of WNT signaling exist including, a repressor of CTNNB1 which could result in the observed inhibition. A recent study by Farookhi and colleagues [46] demonstrated that overexpression of WNT2 in the DC3 rat granulosa cell line led to accumulation of CTNNB1 but not activation of CTNNB1/TCF transcription likely as a consequence of Chibby (CBY1) interaction and suppression of CTNNB1. Additionally, we evaluated Forkhead box protein O1 (*Foxo1*) mRNA expression and demonstrate that *Foxo1* expression was increased by WNT3A alone and in combination with FSH (data not shown). It is interesting that mRNA expression of *Foxo1*, a repressor of folliculogenesis and steroidogenesis [47], is induced in response to WNT3A stimulation. This upregulation in response to stimulation of the canonical WNT pathway may provide another possible mechanism responsible for the inhibitory effect of WNT on FSH mediated gene expression. However, we cannot exclude other signals that may be capable of affecting the steroidogenic enzymes and subsequent hormone production.

In conclusion, FSH stimulation of key steroidogenic enzymes and differentiation factors is negatively regulated by the presence of WNT3A in primary rat granulosa cells. Though the exact molecular nature of the inhibitory effect remains unclear we suggest that FSH regulation of WNT expression sets a negative feedback loop to ensure CTNNB1 remains controlled in an effort to safeguard against overexpression of TCF responsive genes which would result in severe negative consequences. Future studies are required to determine if WNT is acting indirectly via *Axin2*, or through one of several repressor molecules.
